# Salvianolic Acid B Attenuates Iopromide-Induced Renal Tubular Epithelial Cell Injury by Inhibiting the TLR4/NF-*κ*B/NLRP3 Signaling Pathway

**DOI:** 10.1155/2022/8400496

**Published:** 2022-06-26

**Authors:** Ming Xin Pei, Shu Jun Dong, Xin Yue Gao, Ting Luo, Dong Fan, Jun Feng Jin, Xiao Duo Zhao, Yan Ling Chen

**Affiliations:** ^1^Department of Pathophysiology, Zhuhai Campus of Zunyi Medical University, Zhuhai, Guangdong, China; ^2^Department of Pathology, General Hospital of Eastern Theater Command, Nanjing, Jiangsu, China; ^3^Department of Pathology, Suining Central Hospital, Suining, Sichuan, China; ^4^Department of Pathology, Zhuhai Campus of Zunyi Medical University, Zhuhai, Guangdong, China; ^5^Zhongshan School of Medicine, Sun Yat-Sen University, Guangzhou, Guangdong, China

## Abstract

Postcontrast acute kidney injury (PC-AKI) is directly caused by the use of contrast, indicating a clear causal relationship between the contrast and the injury. Salvianolic acid B (Sal B), a water-soluble compound of *Salvia miltiorrhiza*, has a potent anti-inflammatory effect. We conducted a study to explore whether the protective effect of Sal B on iopromide-induced injury in human proximal tubular epithelial cells (HK-2 cells) is related to inhibition of the TLR4/NF-*κ*B/NLRP3 signal pathway. The results showed that 100 *μ*mol/L Sal B counteracted the decrease in cell viability, the increase of ROS and the number of apoptotic cells, and the decrease of mitochondrial membrane potential (ΔΨm) induced by iopromide. Molecular docking analysis showed that Sal B binds TLR4 and NLRP3 proteins. Moreover, 100 *μ*mol/L Sal B also decreased the expression of TLR4, NLRP3, ASC, Caspase-1, IL-18, IL-1*β*, TNF-*α*, p-NF-*κ*B, cleaved caspase-3, and the ratio of Bax/Bcl-2 induced by iopromide. TAK-242, a TLR4 antagonist, was added to further explore the mechanism of Sal B. However, the cotreatment group with TAK-242 and Sal B had no significant difference in cell viability and apoptosis rate compared to the treatment group with TAK-242 or Sal B alone. These results indicated that Sal B can inhibit the TLR4/NF-*κ*B/NLRP3 signal pathway, resulting in the alleviation of iopromide-induced HK-2 cell injury.

## 1. Introduction

With the widespread use of radiology and interventional therapy, acute kidney injury (AKI) after intravenous injection of iodinated contrast media has become a common complication after percutaneous coronary intervention (PCI), with an incidence of 3–15% [1]. Although PC-AKI is usually transient and reversible, it remains a serious clinical problem due to its high incidence and associated outcomes [2]. However, there is currently no effective treatment for PC-AKI. The treatment is limited to hydration therapy and statins [3]. Therefore, an in-depth study of the pathogenesis of PC-AKI and seeking more effective prevention and treatment measures are important to patients and clinical work.

Iopromide has been widely used in clinical practice as a new nonionic low-permeability contrast agent. The hypertonic environment produced by iopromide can cause changes in renal hemodynamics, increase the hydrostatic pressure, and reduce the glomerular filtration rate (GFR) [4]. Iopromide can also exert direct cytotoxicity *in vitro* and induce apoptosis of renal tubular epithelial cells by activating the nucleotide-binding oligomerization domain-like receptor family pyrin domain containing 3 (NLRP3) inflammasome [5].

The Toll-like receptor (TLR) family consists of highly conserved glycoprotein receptors that are parts of the body's natural immune system. Toll-like receptor 4 (TLR4) activates transcription factors, nuclear factor *κ*B (NF-*κ*B), and activator protein 1 (AP-1) through the MyD88-dependent pathway, which is also important for the activation and release of cytokines, chemokines, and costimulatory molecules [6]. NF-*κ*B regulates the expression of proinflammatory factors, transcription factors, and other adhesion molecules, whereas tumor necrosis factor-*α* (TNF-*α*) induces inflammation by activating NF-*κ*B or NF-*κ*B kinase. Activation of TLR4 induces NF-*κ*B signal transduction, resulting in increased expression of NLRP3, proinflammatory interleukin pro-IL-1*β*, and pro-IL-18. The NLRP3 inflammasome is mainly composed of NLRP3, adaptor protein ASC (apoptosis-associated speck-like protein containing a caspase activation and recruitment domain), and effector protein cysteine-aspartic acid protease 1 (Caspase-1) [7]. The exogenous stimuli or endogenous risk signals, the NLRP3 receptors, promote inflammasome formation, which leads to Caspase-1 activation. The activated Caspase-1 cleaves pro-IL-1*β* and pro-IL-18 to mature and secrete them into extracellular cells, producing inflammatory responses [8]. The renal tubules of wild-type mice with iohexol-induced AKI were severely damaged, and their serum creatinine levels were significantly higher than that of nlrp3^−/−^ and casp1^−/−^ mice that indicate the activation of NLRP3 inflammasome in iohexol-induced-AKI mice and alleviation of iohexol-induced kidney injury and apoptosis in nlrp3^−/−^ and casp1^−/−^ mice [9]. Therefore, activation of the TLR4/NF-*κ*B/NLRP3 signal pathway plays an important role in the contrast-induced renal tubular injury. Salvianolic acid B (Sal B) has a variety of pharmacological activities, including antioxidant, antimyocardial ischemia, antitumor, and anti-inflammatory, and has protective effects on the heart, brain, kidney, and other organs [10–12]. Sal B alleviated iohexol-induced renal injury by activating the PI3K/Akt/Nrf2 pathway [13]. In addition, Sal B reduced LPS-stimulated H9c2 cell injury by inhibiting the TLR4/NF-*κ*B/NLRP3 signaling pathway [14]. Our previous study also confirmed that Sal B can inhibit iopromide-induced human proximal tubular epithelial cell (HK-2) injury. However, the specific mechanism needs to be studied [15].

The TLR4/NF-*κ*B/NLRP3 signaling pathway has been a research hotspot in recent years, but the mechanism of Sal B inhibiting the TLR4/NF-*κ*B/NLRP3 signaling pathway in PC-AKI has not been reported. Therefore, in this study, we used iopromide to establish an *in vitro* model of PC-AKI and investigate the protective effect of Sal B to provide an experimental basis for the prevention and treatment of PC-AKI.

## 2. Materials and Methods

### 2.1. Cell Culture and Treatment

Human proximal tubular epithelial cells (HK-2; American Type Culture Collection, Rockville, MD, USA) were donated by Professor Weidong Wang, Sun Yat-sen University. HK-2 cells were cultured in Dulbecco's modified eagle medium/nutrient mixture F12 (DMEM/F12) supplemented with 10% fetal bovine serum and 1% antibiotics (Gibco, USA) and incubated at 37°C with 5% CO_2_. HK-2 cells were treated with different concentrations of iopromide (50, 100, 150, and 200 mgI/mL) for 3 h. According to the results, an *in vitro* model of PC-AKI was established using iopromide at 150 mgI/mL. To evaluate the protective effect of Sal B (Ronghe, Shanghai), HK-2 cells were pretreated with several concentrations of Sal B (10, 50, and 100 *μ*mol/L) for 15 min and then combined with iopromide for 3 h. To investigate the role of the TLR4/NF-*κ*B/NLRP3 signaling pathway in iopromide-induced injury in HK-2 cells, the TLR4 inhibitor TAK-242 (MedChemExpress, USA) was cotreated with iopromide or Sal B.

### 2.2. Cell Counting Kit-8 Assay

HK-2 cells were seeded in a 96-well plate at a density of 1 × 10^4^ cells. After being cultured to 90% confluency, cells received indicated treatments for 3 h. Then, 10% Cell Counting Kit-8 (CCK-8; Dojindo Molecular Technologies, Japan) prepared in serum-free medium was added to each well (100 *μ*L), followed by incubation at 37°C in a 5% CO_2_ atmosphere for 2 h. The absorbance of the cells was measured at 450 nm using a microplate reader (Thermo Fisher Scientific, USA).

### 2.3. 4′,6-Diamidino-2-Phenylindole Staining

HK-2 cells were seeded in a 6-well plate, grown to 90% confluency, and received indicated treatments for 3 h. The original medium was aspirated, and then 4′,6-diamidino-2-phenylindole (DAPI) staining solution (1 *μ*g/L; Sigma, USA) prepared in methanol (1 mL) was then added to each well, followed by incubation at 37°C in a 5% CO_2_ atmosphere for 20 min. The cells were washed three times with phosphate-buffered saline (PBS; Gibco, USA) and visualized under a fluorescence microscope (Olympus, Japan) at ×400 magnification. The number of apoptotic cells was counted using the ImageJ software.

### 2.4. Detection of Mitochondrial Membrane Potential

HK-2 cells were seeded in a 6-well plate, grown to 90% confluency, and received indicated treatments for 3 h. Then, Then, the cells were washed three times with PBS, treated with Rh123 solution (100 *μ*g/L; Sigma, USA) in serum-free DMEM, and incubated at 37°C in a 5% CO_2_ atmosphere for 15 min. The cells were visualized under the fluorescence microscope at ×200 magnification.

HK-2 cells of the experimental group were inoculated in a 6.0 cm Petri dish, followed by administration of indicated treatments for 3 h (another Petri dish was used to inoculate the HK-2 cells of the blank/control group). The cells were washed three times with PBS, digested with trypsin (without EDTA), and then treated with Rh123 solution (100 *μ*g/L) in serum-free DMEM, followed by incubation at 37°C in a 5% CO_2_ atmosphere for 15 min. Fluorescence was detected using flow cytometry (Partec, Germany) after resuspending the cells in PBS and was analyzed using FCS Express software (version 4.0).

### 2.5. Examination of Intracellular Reactive Oxygen Species Generation

HK-2 cells were seeded in a 6-well plate and received indicated treatments for 3 h when they reached 90% confluency. Then the cells were washed with PBS three times, treated with 2′-7′dichlorofluorescein diacetate (DCFH-DA) solution (100 *μ*g/L; Sigma, USA) in serum-free DMEM, and incubated for 15 min. The cells were visualized under a fluorescence microscope at ×200 magnification.

HK-2 cells were inoculated in a 6.0 cm Petri dish, given the indicated treatments for 3 h. And then, the cells were washed with PBS three times, digested with trypsin, treated with 100 *μ*g/L DCFH-DA solution in serum-free DMEM, and incubated in the dark for 15 min. Fluorescence was detected using flow cytometry after resuspending the cells in PBS and was analyzed using FCS Express software (version 4.0).

### 2.6. Detection of Apoptosis

HK-2 cells were cultured in a 6.0 cm Petri dish, treated until they reached 90% confluency, and incubated at 37°C in a 5% CO_2_ atmosphere. After washing three times with PBS and digesting by trypsin without EDTA, the cells were harvested and centrifuged at 1000 r/min for 3 min. The supernatant was then discarded, and 500 *μ*L binding buffer was added to resuspend HK-2 cells. The cells were treated with 5 *μ*L of FITC-Annexin V at 37°C, 5% CO_2_ for 10 min. Then 5 *μ*L of propidium iodide (PI) (KeyGEN Biotech, Nanjing) was added to the mixture, and the cells were stained for 5 min. PBS (3 mL) was then added to each group. Apoptosis was detected using flow cytometry and analyzed using FCS Express software (version 4.0).

### 2.7. Molecular Docking

Possible targets and pathways of Sal B were detected using molecular docking. TLR4 (PDB ID: 3FXI) and NLRP3 (PDB ID: 6NPY) were obtained from the PCSB protein database (https://www.rcsb.org), and the small molecular chemical structure of Sal B was obtained from the PubChem Compound database (https://www.ncbi.nlm.nih.gov/pccompound), respectively. Molecular docking between Sal B and target proteins (TLR4, NLRP3) was calculated using Molecular Operating Environment 2019 software.

### 2.8. Western Blot Analysis

After the indicated treatment, HK-2 cells were lysed in ice-cold radioimmunoprecipitation assay buffer (Beyotime Biotechnology, Shanghai, China) containing 1% phenylmethanesulfonyl fluoride for 30 min. The samples were centrifuged at 15000 rpm for 30 min, and the concentrations of total protein were quantified using a bicinchoninic acid protein assay kit (Thermo Fisher Scientific, USA). Total protein samples (40 *μ*g) were then separated by 8–12% sodium dodecyl sulfate-polyacrylamide gel electrophoresis and transferred onto a polyvinylidene fluoride membrane. Then the membranes were blocked with 5% fat-free milk for 2 h at room temperature and incubated overnight at 4°C with primary antibodies against TLR4, NLRP3, ASC, Caspase-1, TNF-*α*, p-NF-*κ*B, IL-18, IL-1*β*, Bax, Bcl-2, and cleaved caspase-3 (1 : 1000, CST, USA). After washing with PBS-Tween-20, the membranes were indicated with the secondary antibody (1 : 5000, CST, USA) for 1 h at room temperature. The membranes were visualized using electrochemiluminescence reagents (Millipore, USA) and exposed to an infrared laser scanning-imaging instrument (Analytik Jena, Germany). The target proteins were detected with ImageJ software.

### 2.9. Statistical Analysis

Data are presented as the mean ± standard deviation. Statistical significance between multiple groups were analyzed by one-way analysis of variance, followed by the Fisher's least significant difference post hoc test using SPSS (20.0). *P* < 0.05 was considered to indicate a statistically significant difference.

## 3. Results

### 3.1. Sal B Increased the Cell Viability and Mitigated the Iopromide-Induced Apoptosis, the Bax/Bcl-2 Ratio, and the Protein Expression of Cleaved Caspase-3

**Figure 1:**
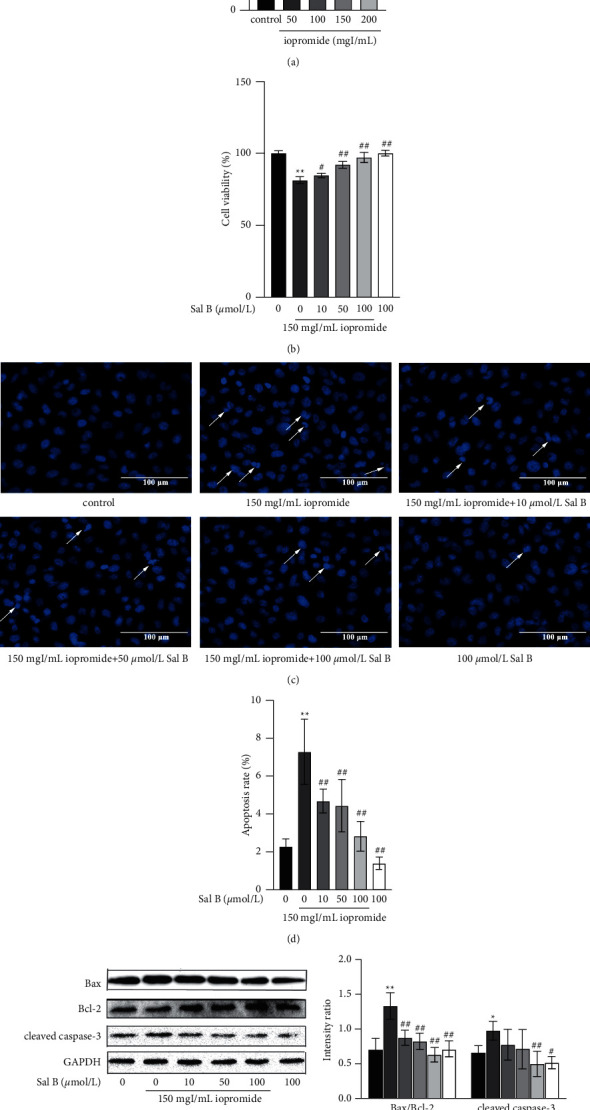
Effect of iopromide and Sal B in HK-2 cell viability. (a) Several doses of iopromide were found to cause a significant decrease in cell viability *n* = 5. Data are the mean ± SD. ^*∗*^*P* < 0.05, ^*∗∗*^*P* < 0.01 vs. control group. (b) Different doses of Sal B were found to reduce HK-2 cells against iopromide-induced injury. *n* = 5. Data are the mean ± SD. ^*∗∗*^*P* < 0.01 vs. control group; ^#^*P* < 0.05, ^##^*P* < 0.01 vs. 150 mgI/mL iopromide group. Several concentrations of Sal B counteracted iopromide-induced apoptosis, Bax/Bcl-2, and cleaved caspase-3 protein levels in HK-2 cells. (c, d) Sal B counteracted the iopromide-induced apoptosis. *n* = 6. Data are the mean ± SD. ^*∗∗*^*P* < 0.01 vs. control group; ^##^*P* < 0.01 vs. iopromide group. (e) The protein levels of Bax/Bcl-2 and cleaved caspase-3 were significantly increased in iopromide-treated cells. These changes were lowered in the 100 *μ*mol/L Sal B group *n* = 3. Data are the mean ± SD. ^*∗*^*P* < 0.05, ^*∗∗*^*P* < 0.01 vs. control group; ^#^*P* < 0.05, ^##^*P* < 0.01 vs. iopromide group.

Different concentrations of iopromide (50, 100, 150, and 200 mgI/mL) were used to detect the cell viability of HK-2 cells using the CCK-8 assay. The cell viability decreased with an increase in the concentration of iopromide (Figure 1(a)). Based on these results, we established a PC-AKI model using 150 mgI/mL iopromide. After adding Sal B (10, 50, and 100 *μ*mol/L), the cell viability increased (Figure 1(b)), indicating that Sal B can mitigate HK-2 cell injury caused by iopromide. After treatment with iopromide, some of the nuclei showed high-density fluorescence and apoptotic characteristics, such as karyopyknosis and karyorrhexis. However, these apoptotic nuclei decreased after treatment with different concentrations of Sal B (Figures 1(c) and 1(d)). Iopromide significantly increased the levels of Bax/Bcl-2 and cleaved caspase-3. Different concentrations of Sal B reduced the ratio of Bax/Bcl-2, while only 100 *μ*mol/L Sal B decreased the expression of cleaved caspase-3 (Figure 1(e)).

### 3.2. Sal B Reduced Iopromide-Induced Reactive Oxygen Species Generation and Increased the Level of Mitochondrial Membrane Potential

**Figure 2:**
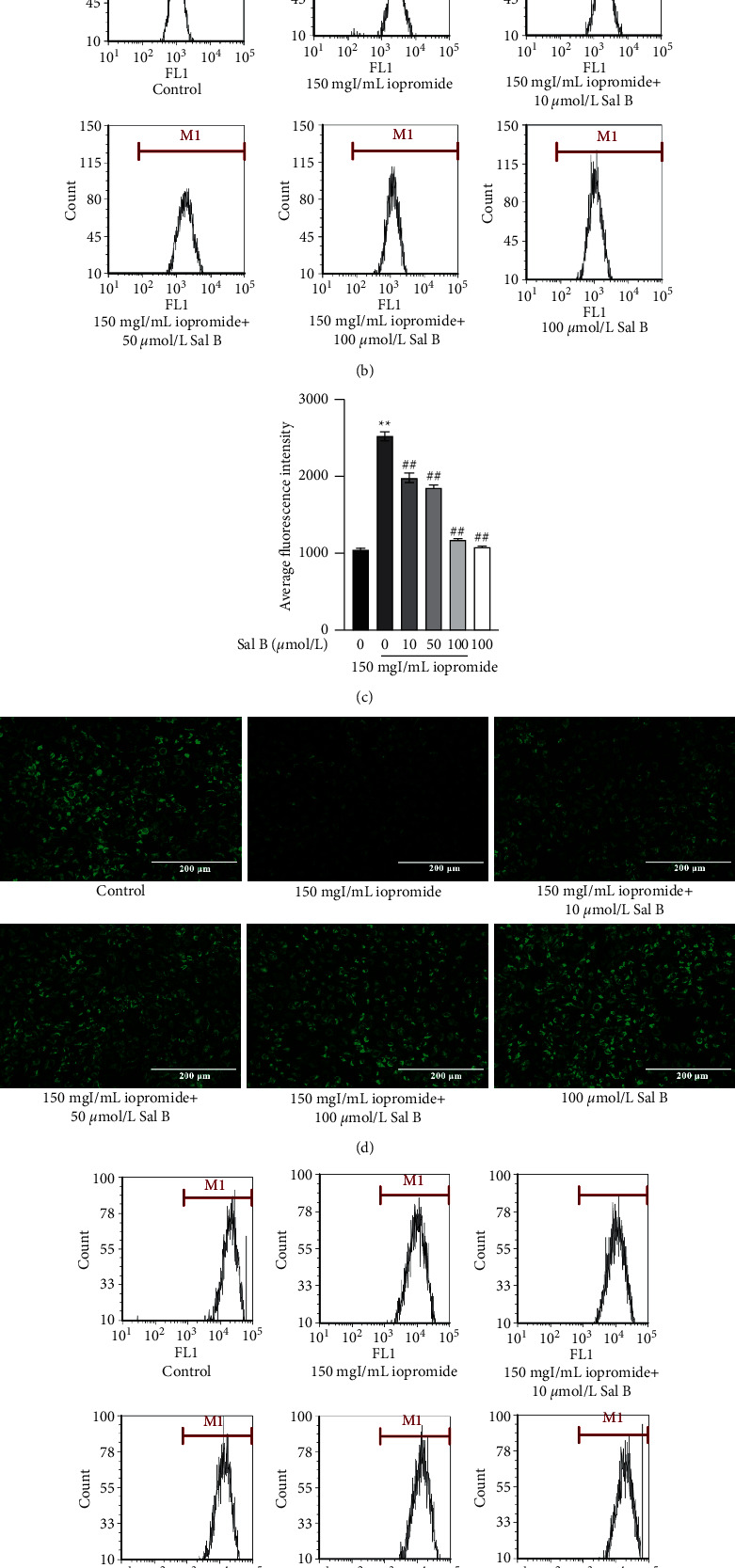
Effect of Sal B on iopromide-induced ROS generation and loss of ΔΨm. (a) Compared with the iopromide-treated group, Sal B lowered the decrease in ROS generation. (b, c) Similar results were obtained by flow cytometry *n* = 3. Data are the mean ± SD. ^*∗∗*^*P* < 0.01 vs. control group; ^##^*P* < 0.01 vs. iopromide group. (d) ΔΨm levels in iopromide-treated cells were lower than those in the control cells. However, Sal B increased the ΔΨm level, indicating that it had a protective effect on iopromide-induced acute kidney injury. (e, f) Similar results were obtained using flow cytometry. *n* = 3. Data are the mean ± SD. ^*∗∗*^*P* < 0.01 vs. control group; ^#^*P* < 0.05, ^##^*P* < 0.01 vs. iopromide group.

When compared with the control group, the fluorescence intensity of the iopromide group was significantly increased; however, the administration of Sal B resulted in a lower level of green fluorescence (Figure 2(a)). Similar results were obtained using flow cytometry. Cells treated with iopromide showed higher mean fluorescence intensity than the control cells. However, the increase in reactive oxygen species (ROS) levels was abolished by treatment with Sal B (Figures 2(b) and 2(c)). Iopromide significantly decreased mitochondrial membrane potential (ΔΨm) levels, as measured using a fluorescence-based assay. Administration of Sal B increased the intensity of green fluorescence, indicating higher levels of ΔΨm (Figure 2(d)). Similar results were obtained using flow cytometry (Figures 2(e) and 2(f)).

### 3.3. Effect of Sal B on Iopromide-Induced Changes in TLR4, NLRP3, ASC, Caspase-1, p-NF-*κ*B, IL-18, IL-1*β*, and TNF-*α* Protein Levels in HK-2 Cells

**Figure 3:**
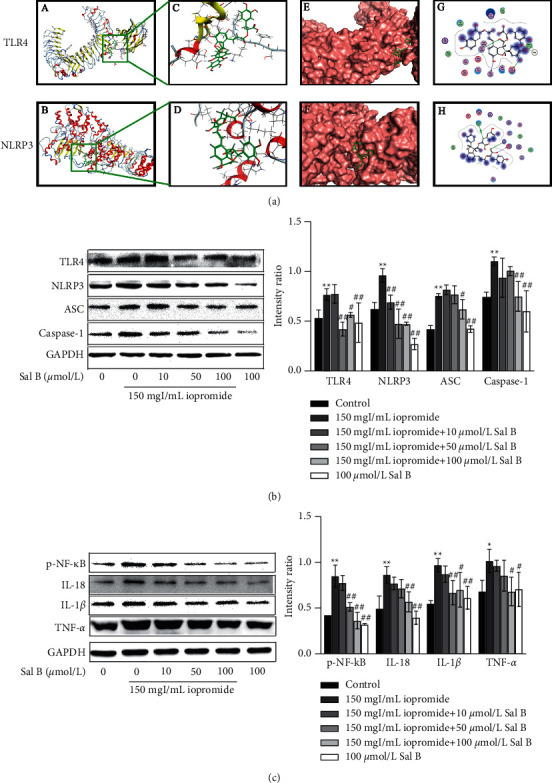
(a) Schematic diagram of the molecular docking between Sal B and target proteins. A, B: conformation of Sal B (green) and target proteins; C, D: the binding sites of Sal B (green) with TLR4 or NLRP3; E, F: schematic diagram of Sal B binding to TLR4 or NLRP3; G, H: the docking amino acid residues of Sal B and TLR4 or NLRP3. Sal B decreased the protein levels of TLR4, NLRP3, ASC, Caspase-1, p-NF-*κ*B, IL-18, IL-1*β*, and TNF-*α*. (b) Iopromide treatment caused a significant increase in TLR4, NLRP3, ASC, and Caspase-1. Sal B counteracted the iopromide-induced increase in these protein levels. *n* = 3. Data are the mean ± SD. ^*∗∗*^*P* < 0.01 vs. control group; ^#^*P* < 0.05, ^##^*P* < 0.01 vs. iopromide group. (c) The protein levels of p-NF-*κ*B, IL-18, IL-1*β*, and TNF-*α* were significantly increased in iopromide-treated cells. These changes were reduced by 100 *μ*mol/L Sal B *n* = 3. Data are the mean ± SD. ^*∗*^*P* < 0.05, ^*∗∗*^*P* < 0.01 vs. control group; ^#^*P* < 0.05, ^##^*P* < 0.01 vs. iopromide group.

Molecular docking analysis showed that Sal B could bind to TLR4 and NLRP3 proteins, with binding energies of −7.64 kcal/mol and −8.56 kcal/mol, respectively. At the same time, the interaction and binding mode of amino acids at the binding site were further investigated (Figure 3(a)). It was found that Sal B can form hydrogen bonds with GLY 389, GLU 369, and SER 392 of TLR4; GLU 280, LYS 216, and PHE 215 of NLRP3. These results suggest that Sal B directly regulates TLR4 or NLRP3 and may play a protective role by inhibiting the TLR4/NF-*κ*B/NLRP3 signaling pathway. Iopromide increased the levels of TLR4, NLRP3, ASC, Caspase-1, p-NF-*κ*B, IL-18, IL-1*β,* and TNF-*α*. We found that 100 *μ*mol/L Sal B reduced the expression of these proteins caused by iopromide (Figures 3(b) and 3(c)).

### 3.4. Effect of Sal B or TAK-242 on Iopromide-Induced Cell Viability

**Figure 4:**
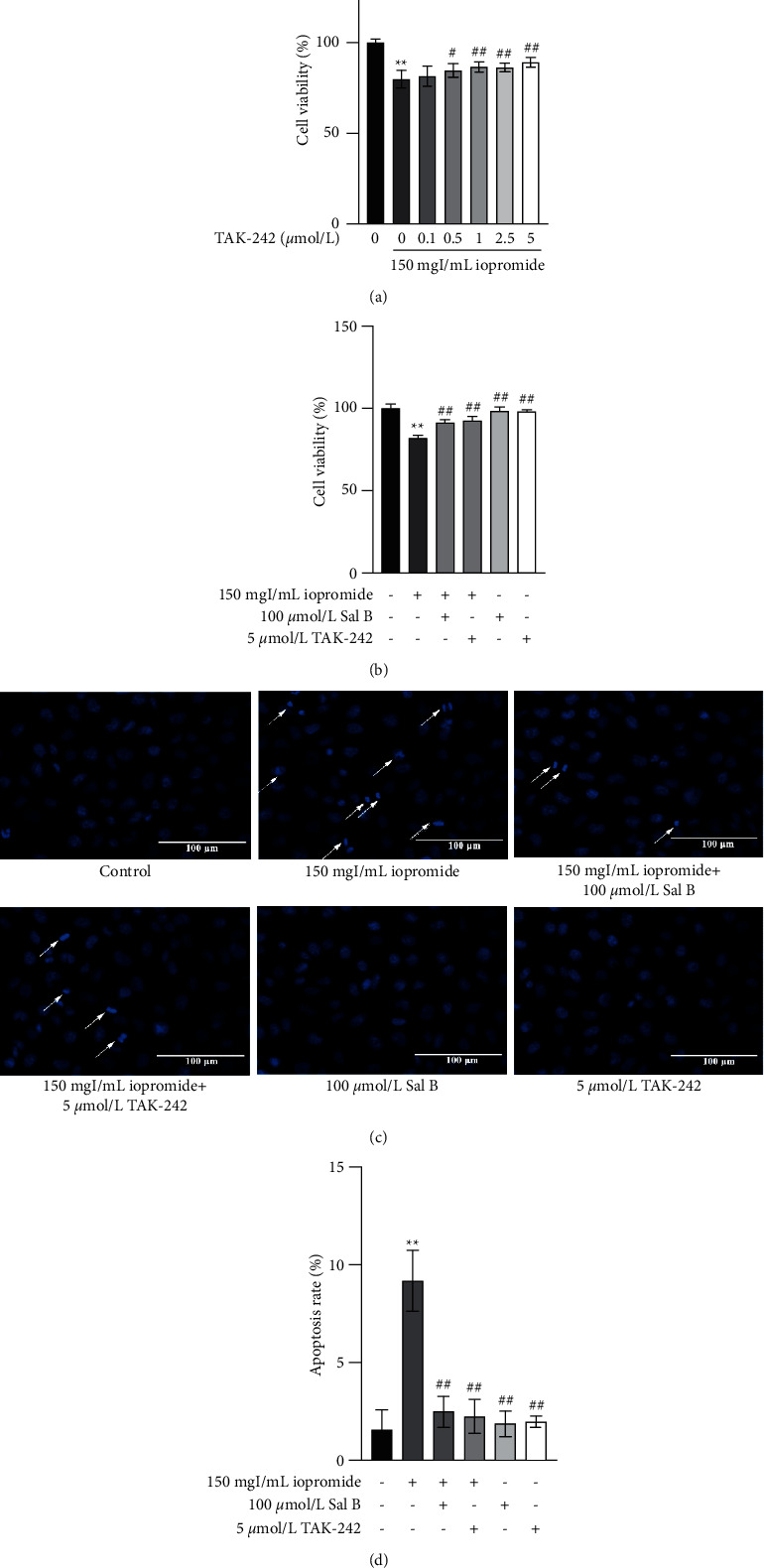
The counteraction of Sal B or TAK-242 on the iopromide-induced apoptosis. (a) TAK-242 was found to protect HK-2 cells against iopromide-induced injury *n* = 5. Data are the mean ± SD. ^*∗∗*^*P* < 0.01 vs. control group; ^#^*P* < 0.05, ^##^*P* < 0.01 vs. iopromide group. (b) Sal B or TAK-242 increased HK-2 cell viability. *n* = 5. Data are the mean ± SD. ^*∗∗*^*P* < 0.01 vs. control group; ^##^*P* < 0.01 vs. iopromide group. (c, d) Sal B or TAK-242 attenuated the iopromide-induced apoptosis. *n* = 6. Data are the mean ± SD. ^*∗∗*^*P* < 0.01 vs. control group; ^##^*P* < 0.01 vs. iopromide group.

To investigate whether the protective role of Sal B is related to the activation of TLR4/NF-*κ*B/NLRP3, we treated cells with TAK-242 (an inhibitor of TLR4). After the addition of different concentrations of TAK-242, the cell viability increased in the TAK-242 groups compared to that in the iopromide group (Figure 4(a)). The protective effect of 5 *μ*mol/L TAK-242 was similar to that of 100 *μ*mol/L Sal B (Figure 4(b)). The nuclear morphology was observed using DAPI staining. The nuclei of the control, Sal B, and TAK-242 groups exhibited low-density blue fluorescence. After 3 h of treatment with iopromide, part of the nucleus showed high-density fluorescence, karyopyknosis, and karyorrhexis. Treatment with Sal B or TAK-242 reduced these apoptotic indicators (Figure 4(c)). The increased number of apoptotic cells was verified by flow cytometry (Figure 4(d)).

### 3.5. Sal B or TAK-242 Reduced Apoptosis Rate, the Ration of Bax/Bcl-2, and the Expression of Cleaved Caspase-3

**Figure 5:**
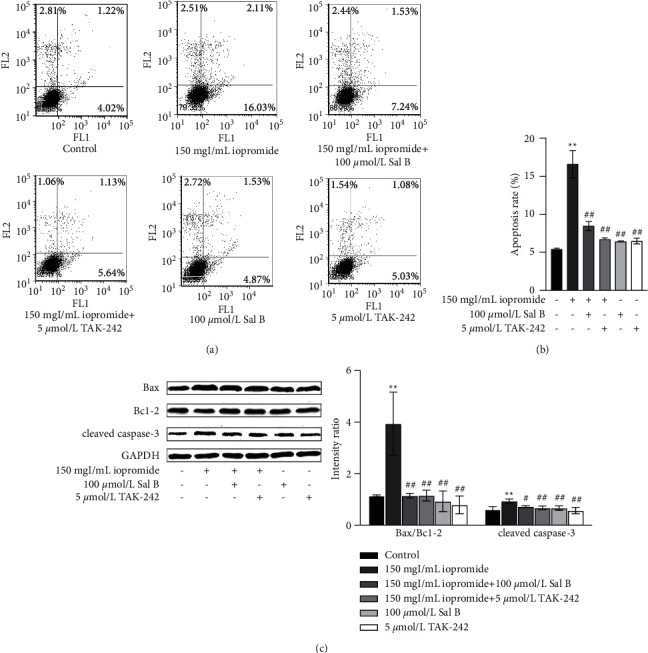
Sal B or TAK-242 counteracted the iopromide-induced apoptosis. (a, b) Sal B or TAK-242 reduced the number of apoptotic cells, as seen in flow cytometry. *n* = 3. Data are the mean ± SD. ^*∗∗*^*P* < 0.01 vs. control group; ^##^*P* < 0.01 vs. iopromide group. (c) Sal B reduced the Bax/Bcl-2 ratio and cleaved caspase-3 *n* = 3. Data are the mean ± SD. ^*∗∗*^*P* < 0.01 vs. control group. ^#^*P* < 0.05, ^##^*P* < 0.01 vs. iopromide group.

The increase in the number of apoptotic cells was verified using flow cytometry. The results showed that the apoptosis rate was significantly increased after treatment with iopromide, while Sal B or TAK-242 reduced apoptosis (Figures 5(a) and 5(b)). Iopromide significantly increased the ratio of Bax/Bcl-2 and the expression of cleaved caspase-3. Treatment with either Sal B or TAK-242 reversed these increases to a similar extent (Figure 5(c)).

### 3.6. Sal B or TAK-242 Protected HK-2 Cells against Iopromide-Induced ROS Generation and Loss of ΔΨm

**Figure 6:**
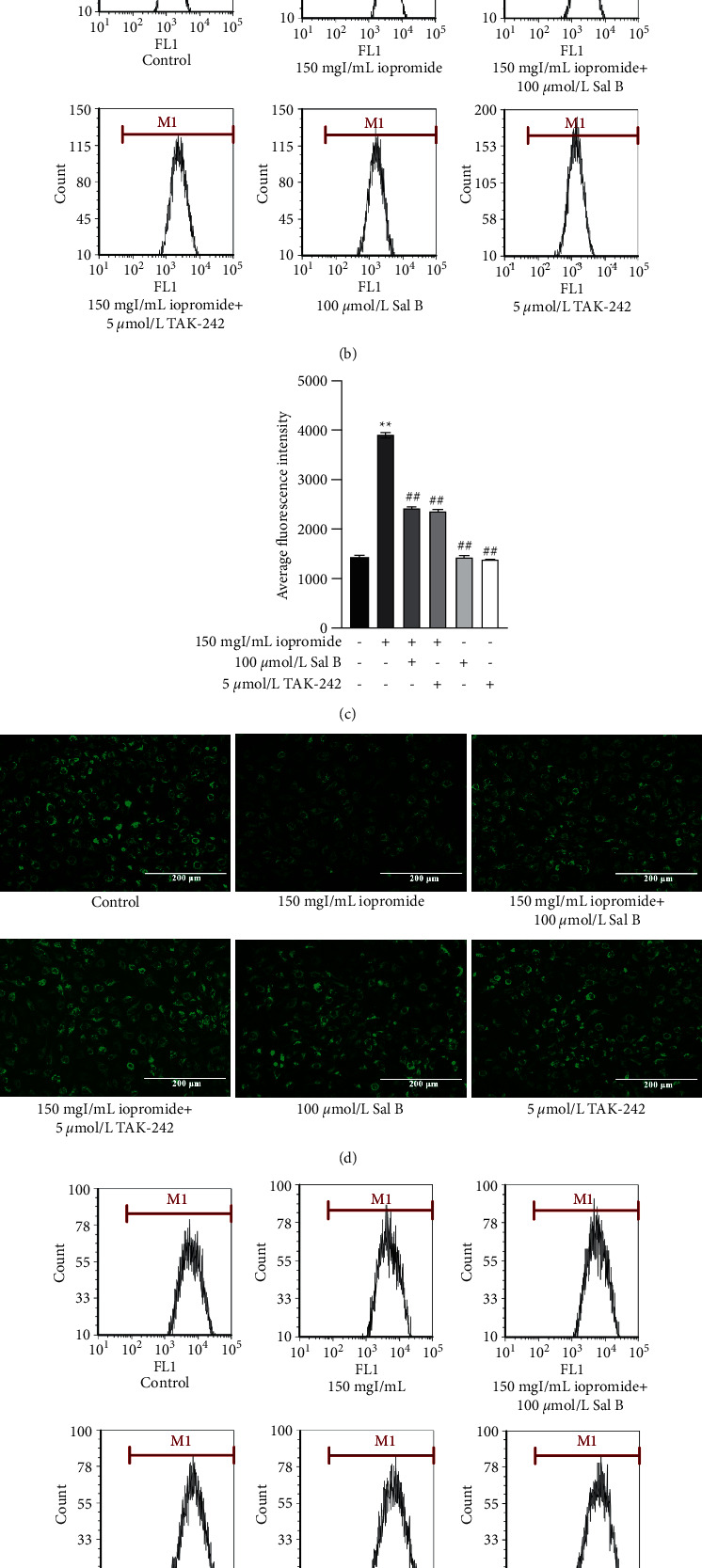
Effect of Sal B or TAK-242 in ROS generation and the levels of ΔΨm. (a) The green fluorescence in iopromide-treated cells was higher than that in control cells. However, Sal B or TAK-242 could reduce the fluorescence intensity. (b, c) Similar results were obtained by flow cytometry *n* = 3. Data are the mean ± SD. ^*∗∗*^*P* < 0.01 vs. control group; ^##^*P* < 0.01 vs. iopromide group. (d) The green fluorescence in iopromide-treated cells was lower than that in control cells. Sal B or TAK-242 treatment increased the fluorescence intensity. (e, f) Similar results were obtained by flow cytometry. *n* = 6. Data are the mean ± SD. ^*∗∗*^*P* < 0.01 vs. control group; ^##^*P* < 0.01 vs. iopromide group.

Treatment of HK-2 cells with 150 mgI/mL iopromide significantly increased ROS generation compared to the controls. Treatment with Sal B or TAK-242 significantly decreased ROS production induced by iopromide (Figure 6(a)). Similar results were obtained using flow cytometry (Figures 6(b) and 6(c)). As illustrated in Figure 6(d), iopromide promoted a significant decrease in ΔΨm compared to the controls, indicating that iopromide may induce mitochondrial damage. However, the loss of ΔΨm was significantly reversed by treatment with Sal B or TAK-242. Similarly, the results of flow cytometry were consistent with the fluorescence microscope (Figures 6(e) and 6(f)).

### 3.7. Sal B or TAK-242 Attenuated Iopromide-Induced Upregulation of TLR4, NLRP3, ASC, Caspase-1, p-NF-*κ*B, IL-18, IL-1*β*, and TNF-*α* Protein Levels

**Figure 7:**
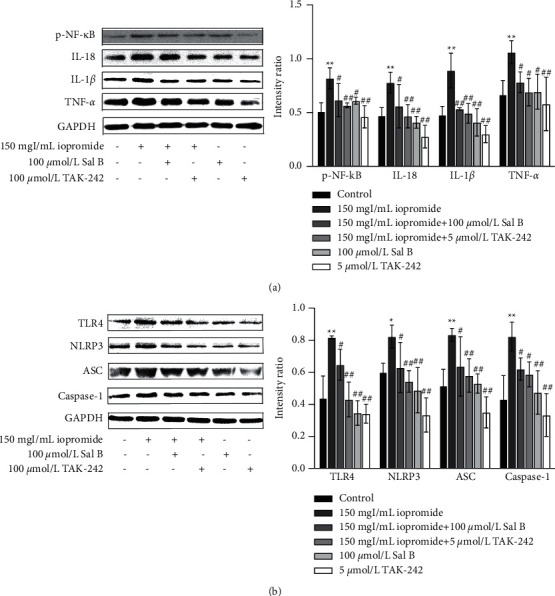
Effect of Sal B or TAK-242 in the levels of TLR4, NLRP3, ASC, Caspase-1, p-NF-*κ*B, IL-18, IL-1*β*, and TNF-*α* proteins induced by iopromide. (a) Sal B reduced the levels of p-NF-*κ*B, IL-18, IL-1*β*, and TNF-*α n* = 3. Data are the mean ± SD. ^*∗∗*^*P* < 0.01 vs. control group; ^#^*P* < 0.05, ^##^*P* < 0.01 vs. iopromide group. (b) Sal B decreased the levels of TLR4, NLRP3, ASC, and Caspase-1; *n* = 3. Data are the mean ± SD. ^*∗*^*P* < 0.05, ^*∗∗*^*P* < 0.01 vs. control group; ^#^*P* < 0.05, ^##^*P* < 0.01 vs. iopromide group.

As shown in Figure 7(a), the expression levels of p-NF-*κ*B, IL-18, IL-1*β*, and TNF-*α* were significantly upregulated following treatment with iopromide compared to the controls. However, the upregulated expression levels of these proteins were suppressed by treatment with Sal B or TAK-242. In addition, Sal B or TAK-242 also reduced the expression of TLR4, NLRP3, ASC, and Caspase-1 (Figure 7(b)), indicating that Sal B may alleviate cell injury by inhibiting the TLR4/NF-*κ*B/NLRP3 signaling pathway.

### 3.8. Effects of Sal B and TAK-242 on Cell Viability and Apoptosis

**Figure 8:**
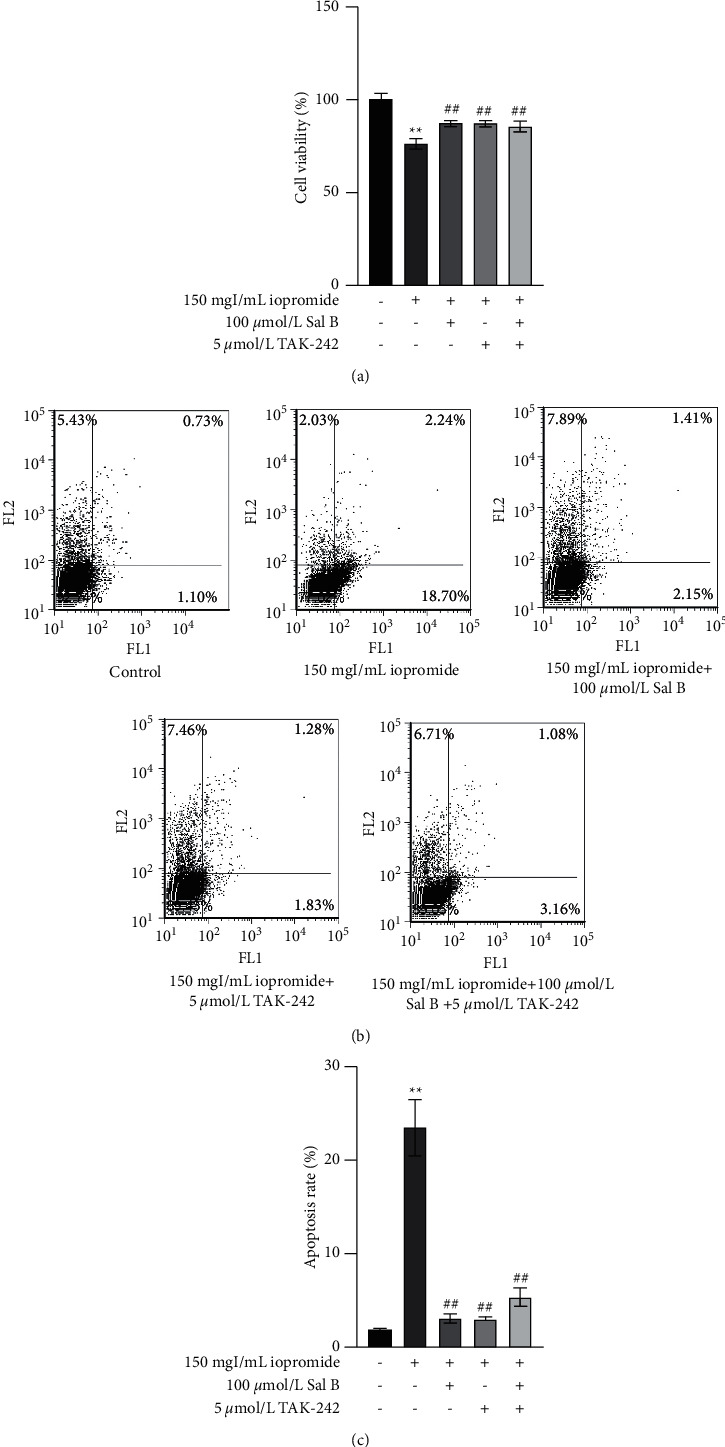
Effects of Sal B and TAK-242 on cell viability and apoptosis. (a) The cotreatment with Sal B and TAK-242 did not further enhance cell viability. *n* = 5. Data are the mean ± SD. ^*∗∗*^*P* < 0.01 vs. control group; ^##^*P* < 0.01 vs. iopromide group. (b) The effect of cotreatment with Sal B and TAK-242 obtained using flow cytometry. *n* = 3. Data are the mean ± SD. ^*∗∗*^*P* < 0.01 vs. control group; ^##^*P* < 0.01 vs. iopromide group.

The addition of Sal B and TAK-242 at the same time showed no significant difference compared to treatment with TAK-242 or Sal B alone (Figure 8(a)), and the flow cytometry results were consistent with the CCK-8 results (Figures 8(b) and 8(c)).

## 4. Discussion

PC-AKI is a common complication of invasive cardiovascular surgery and is considered the third most common cause of hospital-acquired AKI [16, 17]. The toxic effect of iodinated contrast media on proximal renal tubular cells plays an important role in PC-AKI, and massive renal tubular necrosis and interstitial nephritis lead to severe renal failure and significantly increase the risk of death [18]. In this study, we first set different concentrations of iopromide (50, 100, 150, and 200 mgI/mL), following which the HK-2 cells were treated with iopromide for 3 h. The results showed that with the increase in iopromide concentration, the viability of HK-2 cells gradually decreased, and the number of apoptotic cells significantly increased. However, there was no significant difference between the results obtained using 150 mgI/mL and 200 mgI/mL iopromide. Therefore, 150 mgI/mL iopromide was selected for follow-up experiments.

Studies have shown that Sal B has renal protective effects [14]. Our study showed that after treatment with Sal B (10, 50, and 100 *μ*mol/L), HK-2 cell viability was increased and the number of apoptotic cells was decreased significantly, suggesting that Sal B can resist iopromide-induced HK-2 cell injury through antiapoptosis. Molecular docking results also showed that Sal B can stably bind to TLR4 and NLRP3 spontaneously, with binding free energies of −7.64 kcal/mol and −8.56 kcal/mol, respectively. In addition, the protective effect of Sal B against iopromide-induced HK-2 cell injury was similar to that of TAK-242. Therefore, we concluded that Sal B may alleviate iopromide-induced HK-2 cell death by inhibiting the TLR4/NF-*κ*B/NLRP3 signaling pathway. Apoptosis is an autonomous and orderly death of cells controlled by genes [19, 20]. Apoptosis can be divided into two main pathways: exogenous and endogenous [21]. The exogenous pathway refers to the apoptosis process mediated by the death receptor. The membrane receptors involved in apoptosis belong to the tumor necrosis factor (TNF) receptor family, whose activation is mainly dependent on two ligands: TNF and Fas [22]. TNF receptor-associated and Fas-associated death domain proteins bind to their corresponding ligands to recruit the downstream factor pro-caspase-8, which is automatically cleaved to produce active caspase-8, thus initiating apoptosis. Caspase-8 further activates pro-caspase-3 through protein hydrolysis, resulting in cleaved caspase-3, which is ultimately responsible for intracellular protein hydrolysis and apoptosis induction [23–25]. Endogenous apoptosis is characterized by mitochondrial regulation [26]. Cytochrome C binds to apoptotic protease activating factor −1 to form apoptotic bodies, which recruit and activate pro-caspase-9, and then activate downstream caspase-3 and caspase-7 to perform apoptotic reactions [27–29]. The openness of mitochondrial membrane potential (mPTP) is affected by Ca^2+^, ATP, ROS, the Bcl-2 family, and other factors [30, 31]. ROS not only directly regulates the opening of mPTP through oxidation of different sites but also promotes the release of Ca^2+^ from the endoplasmic reticulum to the mitochondria, indirectly regulating the openness of mPTP and resulting in a decrease in ΔΨm [32]. The Bcl-2 family is divided into three groups: antiapoptotic protein Bcl-2, proapoptotic effector proteins Bax and Bak, and BH-3 only domain proteins [33]. When apoptosis is initiated, Bax and Bak are activated and accumulated in the mitochondrial outer membrane (MOM), which changes the mPTP and leads to activation of the caspase cascade and cell death [34–36]. In addition, antiapoptotic proteins Bcl-2 and BH-3 are the only domain proteins that act in MOM, guaranteeing cell survival by inhibiting the activity of proapoptotic proteins [37].

In this study, HK-2 cells in the control group showed uniform low-density blue fluorescence after DAPI staining, whereas some cells showed high-density fluorescence and other apoptotic characteristics after treatment with iopromide for 3 h. Iopromide increased ROS generation, the ratio of Bax/Bcl-2, and cleaved caspase-3 level in HK-2 cells and decreased ΔΨm, indicating that iopromide may decrease ΔΨm through the Bcl-2 family and ROS generation and then induce the apoptosis of HK-2 cells. However, after treatment with Sal B, apoptotic cells, ROS generation, Bax/Bcl-2 ratio, and cleaved caspase-3 levels decreased, whereas ΔΨm increased, suggesting that Sal B may play an antiapoptotic role through the mitochondrial pathway and reduce iopromide-induced cell injury.

The excessive inflammatory response may induce tissue damage through the direct toxic effects of circulating cytokines and chemokines, thicken the glomerular basement membrane, and aggravate renal interstitial fibrosis [38]. There is increasing evidence showing that renal tubular epithelial cells, as specialized immune cells, can mitigate renal injury to some extent by regulating innate and adaptive immune responses [39]. The activation of NLRP3 inflammasome is of great significance in PC-AKI. In the resting state, NLRP3 inflammasome exists in autoinhibitory form, but in response to external stimuli, it is activated and assembled into a mature complex of NLRP3, ASC, and pro-caspase-1. Activated Caspase-1 subsequently lyses pro-IL-1*β* and pro-IL-18 to form mature IL-1*β* and IL-18, which further activates an inflammatory cascade, followed by inflammatory cell infiltration and renal tissue destruction [40]. NLRP3 inflammasome not only mediates the progression of renal disease by activating inflammatory responses in immune cells but also regulates the apoptosis of renal tubular epithelial cells by interacting with mitochondria and mediating the production of ROS and mitochondrial autophagy [39]. Our results showed that iopromide can activate the NLRP3 inflammasome in HK-2 cells, increase the expression of NLRP3, ASC, and Caspase-1, and activate IL-1*β* and IL-18 to produce extracellular inflammatory responses. However, the NLRP3 inflammasome was inhibited, and the expressions of NLRP3, ASC, Caspase-1, IL-1*β,* and IL-18 were decreased in HK-2 cells after treating with Sal B. These results suggested that HK-2 cell injury induced by iopromide is related to the activation of the NLRP3 inflammasome, and Sal B can alleviate the injury by inhibiting the activation of NLRP3 inflammasome.

In response to various invasive pathogens and tissue damage, the innate immune system activates pattern recognition receptors (PRR) to initiate inflammatory responses [41]. Currently, identified members of the PRR family include Toll-like receptor (TLR), c-type lectin receptor (CLR), retinoic acid-inducible gene-1-like receptor (RLR), nucleotide-binding oligomerization domain- (NOD-) like receptor (NLR), and secretory proteins [42]. Toll-like receptor 4 (TLR4), the first identified mammalian Toll-like protein, plays an important role in initiating innate immune response [43]. TLR4 can be linked to TLR2 through the connector molecule MyD88, resulting in the activation of the transcription factor, nuclear factor *κ*B (NF-*κ*B) and AP1, and the release of TNF-*α*, IL-1*β*, IL-6, and IL-8 [6]. Activation of TLR4 induces NF-*κ*B signal transduction, leading to increased expression of NLRP3, pro-IL-1 *β,* and pro-IL-18. Studies have shown that the inflammatory cytokines such as IL-6 and TNF-*α* were significantly increased in rats treated with loversol, resulting in renal dysfunction, apoptosis of renal tubular epithelial cells, and serious renal tubular damage, while antithrombin III could reduce renal injury by inhibiting inflammation [44]. In our study, the expression levels of TLR4, NF-*κ*B, NLRP3, ASC, Caspase-1, IL-18, IL-1*β*, and TNF-*α* in HK-2 cells were significantly increased after administration of iopromide. These results suggest that iopromide may further activate the NLRP3 inflammasome through the TLR4/NF-*κ*B/NLRP3 signaling pathway, resulting in the injury of HK-2 cells and that Sal B treatment decreased the expression of these proteins.

**Figure 9:**
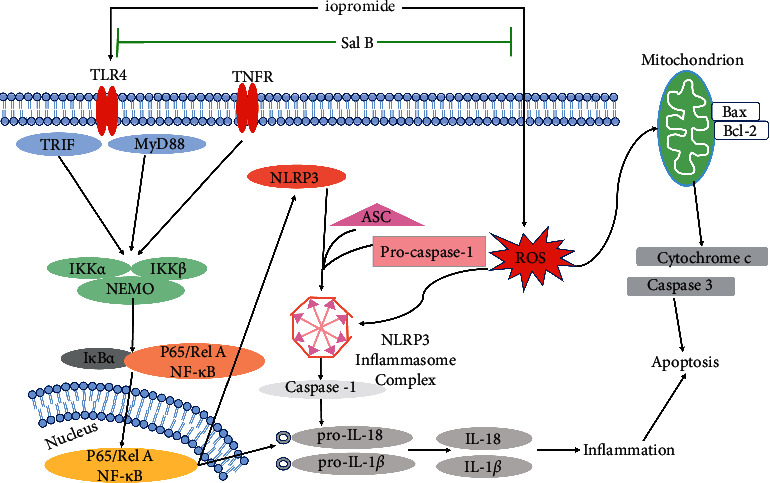
Effect of a schematic diagram showing the role of Sal B in the attenuation of iopromide-induced HK-2 cell injury by inhibiting the TLR4/NF-*κ*B/NLRP3 signaling pathway.

To further investigate the effects of the TLR4/NF-*κ*B/ NLRP3 signaling pathway on HK-2 cells, these cells were treated with the TLR4 inhibitor TAK-242. The results showed that after treatment with TAK-242, HK-2 cell viability and ΔΨm levels increased, whereas apoptosis rate, ROS generation, expression of TLR4 and NF-*κ*B, NLRP3 inflammasome-related components, TNF-*α*, IL-1*β*, and IL-18 decreased significantly. In addition, the protective effect of Sal B is similar to that of TAK-242. These results suggest that iopromide-induced HK-2 cell injury is closely related to activating the TLR4/NF-*κ*B/NLRP3 signaling pathway. The cotreatment group with TAK-242 and Sal B had no significant difference in cell viability and apoptosis rate compared to the treatment group with TAK-242 or Sal B alone. The use of TAK-242 can inhibit the TLR4/NF-*κ*B/NLRP3 signaling pathway to produce a protective effect. If Sal B acts on other pathways at the same time, the protection effect may be more obvious. Double inhibition did not enhance the protection of HK-2 cells, which further verified that Sal B may inhibit the TLR4/NF-*κ*B/NLRP3 signaling pathway. The schematic diagram of this study is shown in Figure 9 [8, 44].

## 5. Conclusion

The present study investigated the activation of the TLR4/NF-*κ*B/NLRP3 signaling pathway in iopromide-induced HK-2 cell injury. We found that Sal B attenuates iopromide-induced HK-2 cell injury, reduces apoptosis and ROS generation, enhances ΔΨm level, and inhibits TLR4/NF-*κ*B/NLRP3 signaling pathway.

## Data Availability

The data that support the findings of this study are available from the corresponding author.

## References

[1] Wang C., Li G., Liang X. (2020). Predictive value of fibrinogen-to-albumin ratio for post-contrast acute kidney injury in patients undergoing elective percutaneous coronary intervention. *Medical Science Monitor*.

[2] Azzalini L., Spagnoli V., Ly H. Q. (2016). Contrast-induced nephropathy: from pathophysiology to preventive strategies. *Canadian Journal of Cardiology*.

[3] Zhang F., Lu Z., Wang F. (2020). Advances in the pathogenesis and prevention of contrast-induced nephropathy. *Life Sciences*.

[4] Mamoulakis C., Fragkiadoulaki I., Karkala P. (2019). Contrast-induced nephropathy in an animal model: evaluation of novel biomarkers in blood and tissue samples. *Toxicology Reports*.

[5] Chen Y. H., Fu Y. C., Wu M. J. (2019). Does resveratrol play a role in decreasing the inflammation associated with contrast induced nephropathy in rat model?. *Journal of Clinical Medicine*.

[6] Ciesielska A., Matyjek M., Kwiatkowska K. (2021). TLR4 and CD14 trafficking and its influence on LPS-induced pro-inflammatory signaling. *Cellular and Molecular Life Sciences*.

[7] Biasizzo M., Kopitar-Jerala N. (2020). Interplay between NLRP3 inflammasome and autophagy. *Frontiers in Immunology*.

[8] Seoane P. I., Lee B., Hoyle C. (2020). The NLRP3-inflammasome as a sensor of organelle dysfunction. *Journal of Cell Biology*.

[9] Lin Q., Li S., Jiang N. (2021). Inhibiting NLRP3 inflammasome attenuates apoptosis in contrast-induced acute kidney injury through the upregulation of HIF1A and BNIP3-mediated mitophagy. *Autophagy*.

[10] Lin C., Liu Z., Lu Y. (2016). Cardioprotective effect of salvianolic acid B on acute myocardial infarction by promoting autophagy and neovascularization and inhibiting apoptosis. *Journal of Pharmacy and Pharmacology*.

[11] Katary M. A., Abdelsayed R., Alhashim A., Abdelhasib M., Elmarakby A. A. (2019). Salvianolic acid B slows the progression of breast cancer cell growth via enhancement of apoptosis and reduction of oxidative stress, inflammation, and angiogenesis. *International Journal of Molecular Sciences*.

[12] Wang B. A.-O. X., Sun J., Shi Y., Le G. (2017). Salvianolic acid B inhibits high-fat diet-induced inflammation by activating the Nrf2 pathway. *Journal of Food Science*.

[13] Tongqiang L., Shaopeng L., Xiaofang Y. (2016). Salvianolic acid B prevents iodinated contrast media-induced acute renal injury in rats via the PI3K/Akt/Nrf2 pathway. *Oxidative Medicine and Cellular Longevity*.

[14] Hu Y., Li Q., Pan Y., Xu L. (2019). Sal B alleviates myocardial ischemic injury by inhibiting TLR4 and the priming phase of NLRP3 inflammasome. *Molecules*.

[15] Dong S. J., Gao X. Y., Pei M. X. (2021). Effects and mechanism of salvianolic acid B on the injury of human renal tubular epithelial cells induced by iopromide. *Frontiers in Pharmacology*.

[16] Sekiguchi H., Ajiro Y., Uchida Y. (2018). Contrast-induced nephropathy and oxygen pretreatment in patients with impaired renal function. *Kidney International Reports*.

[17] Palli E., Makris D., Papanikolaou J., Garoufalis G., Zakynthinos E. (2014). Contrast-induced nephropathy in aged critically ill patients. *Oxidative Medicine and Cellular Longevity*.

[18] Zhang Z., Shao X., Jiang N. (2018). Caspase-11-mediated tubular epithelial pyroptosis underlies contrast-induced acute kidney injury. *Cell Death and Disease*.

[19] Xu X., Lai Y., Hua Z. C. (2019). Apoptosis and apoptotic body: disease message and therapeutic target potentials. *Bioscience Reports*.

[20] Kerr J. F. (2002). History of the events leading to the formulation of the apoptosis concept. *Toxicology*.

[21] Bock F. J., Tait S. W. G. (2020). Mitochondria as multifaceted regulators of cell death. *Nature Reviews Molecular Cell Biology*.

[22] Liedtke C., Trautwein C. (2012). The role of TNF and Fas dependent signaling in animal models of inflammatory liver injury and liver cancer. *European Journal of Cell Biology*.

[23] Mandal R., Barron J. C., Kostova I., Becker S., Strebhardt K. (2020). Caspase-8: the double-edged sword. *Biochimica et Biophysica Acta (BBA)—Reviews on Cancer*.

[24] Wang M., Su P. (2018). The role of the Fas/FasL signaling pathway in environmental toxicant-induced testicular cell apoptosis: an update. *Systems Biology in Reproductive Medicine*.

[25] Yi F., Frazzette N., Cruz A. C., Klebanoff C. A., Siegel R. M. (2018). Beyond cell death: new functions for TNF family cytokines in autoimmunity and tumor immunotherapy. *Trends in Molecular Medicine*.

[26] Cosentino K., Garcia-Saez A. J. (2014). Mitochondrial alterations in apoptosis. *Chemistry and Physics of Lipids*.

[27] Santucci R., Sinibaldi F., Cozza P., Polticelli F., Fiorucci L. (2019). Cytochrome c: an extreme multifunctional protein with a key role in cell fate. *International Journal of Biological Macromolecules*.

[28] Kalpage H. A., Bazylianska V., Recanati M. A. (2019). Tissue specific regulation of cytochrome *c* by post translational modifications: respiration, the mitochondrial membrane potential, ROS, and apoptosis. *The FASEB Journal*.

[29] Guerra-Castellano A., Marquez I., Perez-Mejias G., Diaz-Quintana A., De la Rosa M. A., Diaz-Moreno I. (2020). Post-translational modifications of cytochrome c in cell life and disease. *International Journal of Molecular Sciences*.

[30] Li Y., Sun J., Wu R. (2020). Mitochondrial mptp: a novel target of ethnomedicine for stroke treatment by apoptosis inhibition. *Frontiers in Pharmacology*.

[31] Kinnally K. W., Peixoto P. M., Ryu S. Y., Dejean L. M. (2011). Is mPTP the gatekeeper for necrosis, apoptosis, or both?. *Biochimica et Biophysica Acta (BBA)—Molecular Cell Research*.

[32] Zhang J., Wang X., Vikash V. (2016). ROS and ROS-mediated cellular signaling. *Oxidative Medicine and Cellular Longevity*.

[33] Ugarte-Uribe B., Garcia-Saez A. J. (2017). Apoptotic foci at mitochondria: in and around bax pores. *Philosophical Transactions of the Royal Society B: Biological Sciences*.

[34] Edlich F. (2018). BCL-2 proteins and apoptosis: recent insights and unknowns. *Biochemical and Biophysical Research Communications*.

[35] Flores-Romero H., Ros U., Garcia-Saez A. J. (2020). Pore formation in regulated cell death. *The EMBO Journal*.

[36] Luo X., O’Neill K. L., Huang K. (2020). The third model of Bax/Bak activation: a Bcl-2 family feud finally resolved?. *F1000Research*.

[37] Lindsay J., Esposti M. D., Gilmore A. P. (2011). Bcl-2 proteins and mitochondria—specificity in membrane targeting for death. *Biochimica et Biophysica Acta (BBA)—Molecular Cell Research*.

[38] Cantaluppi V., Quercia A. D., Dellepiane S., Ferrario S., Camussi G., Biancone L. (2014). Interaction between systemic inflammation and renal tubular epithelial cells. *Nephrology Dialysis Transplantation*.

[39] Qi R., Yang C. (2018). Renal tubular epithelial cells: the neglected mediator of tubulointerstitial fibrosis after injury. *Cell Death and Disease*.

[40] Sharma M., De Alba E. (2021). Structure, activation and regulation of NLRP3 and AIM2 inflammasomes. *International Journal of Molecular Sciences*.

[41] Satoh T., Akira S. (2016). Toll-like receptor signaling and its inducible proteins. *Microbiology Spectrum*.

[42] Takeuchi O., Akira S. (2010). Pattern recognition receptors and inflammation. *Cell*.

[43] Kuzmich N. N., Sivak K. V., Chubarev V. N., Porozov Y., Savateeva-Lyubimova T., Peri F. (2017). TLR4 signaling pathway modulators as potential therapeutics in inflammation and sepsis. *Vaccines*.

[44] Lu Z., Cheng D., Yin J. (2017). Antithrombin III protects against contrast-induced nephropathy. *EBioMedicine*.

